# Association Between Increased Elbow Carrying Angle and Lateral Epicondylitis

**DOI:** 10.7759/cureus.22981

**Published:** 2022-03-09

**Authors:** Levent F Umur, Serkan Surucu

**Affiliations:** 1 Orthopaedics and Traumatology, Acibadem Kadikoy Hospital, Istanbul, TUR; 2 Orthopaedics/Clinical Research, University of Missouri–Kansas City, Kansas City, USA

**Keywords:** tendinopathy, tennis elbow, lateral epicondylitis, extensor carpi radialis brevis, elbow carrying angle

## Abstract

Introduction

The goal of this study was to ascertain the effect of increased elbow carrying angle (ECA) in lateral epicondylitis (LE) development.

Materials and methods

This retrospective study involved a total of 62 participants between January and December 2021, of whom 29 were diagnosed with LE. Physical examinations and elbow radiographs of the patients were reviewed retrospectively. ECAs were measured with the elbow fully extended and the forearm fully supinated on anteroposterior elbow radiographs. Two experienced orthopedic surgeons separately evaluated the values on the radiograph.

Results

This study involved 62 individuals, of which 55.4% are female and 44.6% are male. The mean age of the patients was 45.45 ± 4.77 years (range, 40-69 years), and the mean body mass index (BMI) was 28.1 ± 3.8 kg/m^2^ (range, 19-34 kg/m^2^). There were significant differences in elbow carrying angle between the LE group and the control group (p < 0.05). Also, there was a significant correlation between the LE side and the dominant side (p < 0.05).

Conclusion

Increased ECA is associated with increased incidence of LE and may contribute to its etiology by elevating extensor carpi radialis brevis (ECRB) tendon tension and rerouting it, resulting in increased abrasive and pressurizing forces.

## Introduction

Lateral epicondylitis (LE) is an overuse injury induced by an overload of extensor tendons at the origin of the extensor carpi radialis brevis (ECRB) tendon [1]. Repetitive microtraumas, cyclic overloading of the forearm extensor muscles (wrist extension and grip), and the use of vibrating heavy machinery are all potential causes [2]. Apart from these functional explanations, anatomical variables resulting in lateral wear have been identified as possible pathogenetic factors [3].

The elbow carrying angle (ECA) is the angle made by the arm and forearm in the anatomical (extension-supination) position of the upper extremity. It is determined radiologically or clinically by the angle formed by the humeral and the forearm axis. The normal range for ECA is 5°-15°, which is greater in females than in males [4]. Values outside of this range may result in functional impairment and/or clinical features such as neuropathies, arthritis, and range of motion loss. Numerous studies have investigated the association between upper extremity injuries and ECA from a variety of perspectives [5,6], but to our knowledge, no studies have evaluated isolated ECA in the absence of other possible etiologic factors in patients with LE.

The purpose of this study was to determine the role of ECA in the development of LE. Our hypothesis was that by altering the course of the ECRB during forearm and wrist movement from supination-flexion to pronation-extension, it would remain under increased tension and pressure as a result of the increased carrying angle, hence increasing a risk factor for LE.

## Materials and methods

**Figure 1:**
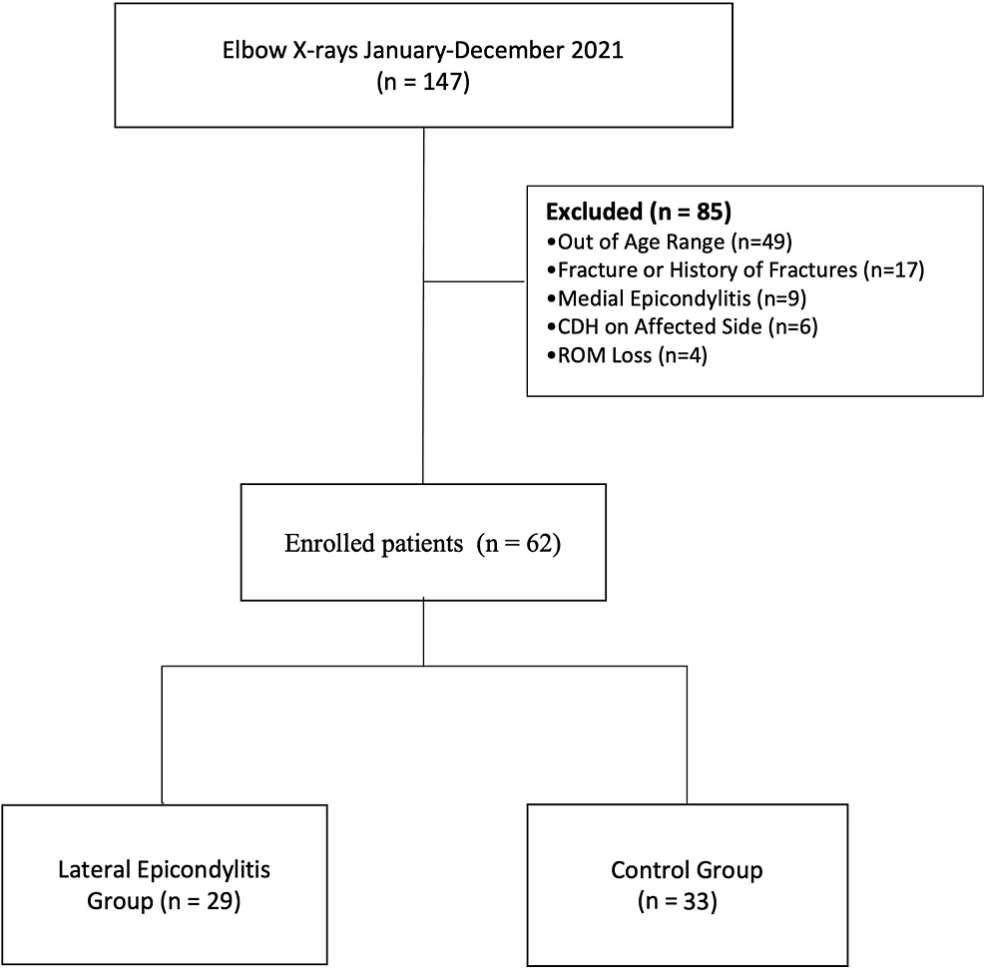
Flowchart of the study. CDH: cervical disc hernia, ROM: range of motion

Between January and December 2021, a total of 62 out of 147 patients with elbow X-rays were enrolled in this retrospective study, of whom 29 were diagnosed with lateral epicondylitis. Retrospectively, the physical examination and elbow radiographs of the patients were evaluated. Patients with a history of surgery or fracture on the affected side, under the age of 18 and over 60, who are tennis players, with a history of cervical disc pathologies, and with loss in the range of motion (passive forearm pronation > 80°, supination > 85°, passive wrist flexion > 85°, and extension > 70°) were excluded. For the control group, the same exclusion criteria and, additionally, a negative history for LE were used, and the remaining 33 patients with elbow X-rays for any other reasons were included (Figure 1). Demographic data and ECA values between the two groups were evaluated.

**Figure 2:**
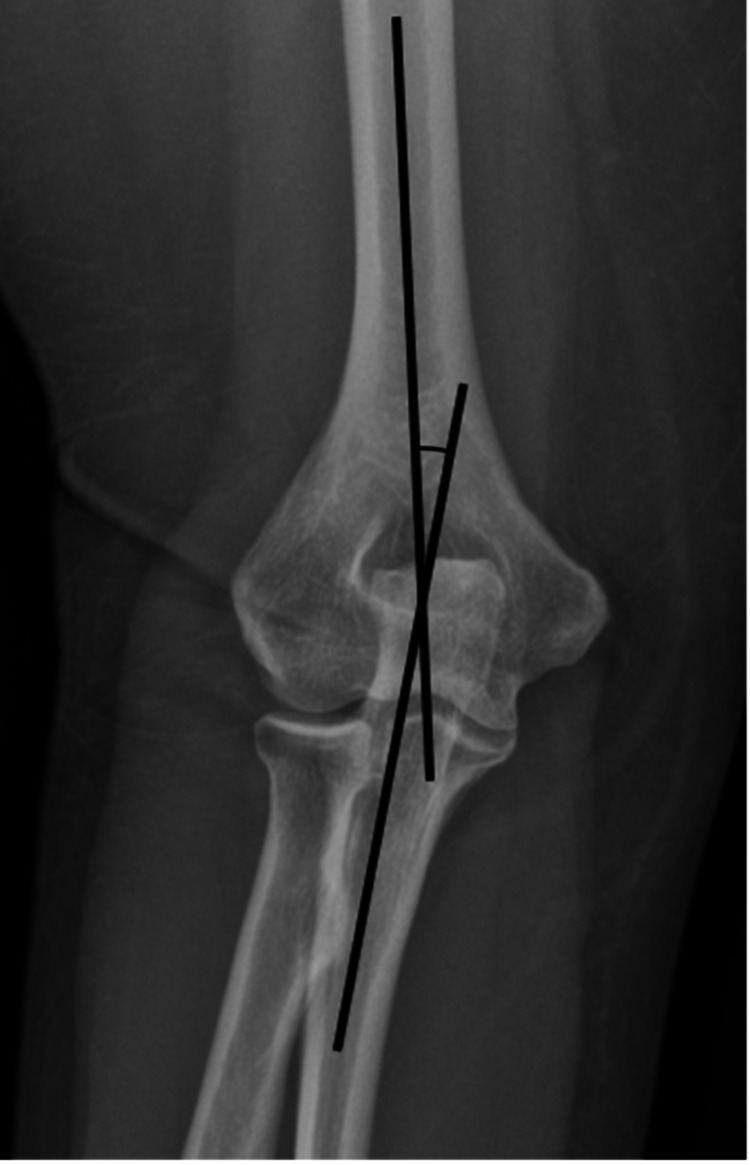
Measurement of the elbow carrying angle on anteroposterior elbow radiographs.

ECAs were determined with the elbow extended and the forearm supinated on anteroposterior elbow radiographs. Radiographs with obliquity and rotation were excluded. On the radiograph, the carrying angle was measured using the method described by Alsubael and Hegazy [4]. Two midpoints on the distal humerus were noted on the radiograph, one at the distal metaphysis and the other in the distal third of the diaphysis. On the ulna, two midpoints were identified: one at the level of radial tuberosity and another at the ulna’s most proximal ossification. A line was drawn between the matching bone’s points, and the software calculated the angle between the two lines (Figure 2).

Measurements were made using the Infinitt PACS system (INFINITT Healthcare, Seoul, South Korea) to view radiographs. The values on the radiograph were measured by two experienced orthopedic surgeons. Two weeks later, the same surgeons repeated the measurements. Inter- and intraobserver reliability were measured for radiographic measurements using intraclass correlation coefficients (ICCs) calculated from two sets of repeat measurements on a sample of 62 radiographs. The following scores were used: ICC greater than 0.80 indicates excellent; 0.70-0.80 indicates very good; 0.60-0.70 indicates good; 0.40-0.60 indicates fair; and 0.40 indicates poor.

There is a distribution of demographic data and descriptive statistics about age. The variation in ECA measurement between study groups was investigated using the T-test in independent groups. Chi-square analysis was used to determine the association between the injured and dominant sides. The SPSS version 20.0 (IBM Corporation, Armonk, New York, USA) software was used to conduct analyses at a 5% level of error.

## Results

Sixty-two individuals enrolled in the study. The lateral epicondylitis group comprised 29 of 62 patients, whereas the control group consisted of 33 of 62 patients. Females account for 55.4%, and males account for 44.6%. The patients’ mean age was 45.45 ± 4.77 years (range, 40-69 years), and their mean body mass index (BMI) was 28.1 ± 3.8 kg/m^2^ (range, 19-34 kg/m^2^). Between the two groups, there were no significant differences in age, gender, BMI, or dominance (p > 0.05).

The rate of those with lateral epicondylitis on the right side is 58.6%, while the rate of those with lateral epicondylitis on the left side is 41.4%. While 72.2% of the individuals with the dominant side on the right had lateral epicondylitis on that side, 63.6% of those with the dominant side on the left had lateral epicondylitis on that side. There was a significant correlation between the lateral epicondylitis side and the dominant side (p < 0.05) (Table 1).

**Table 1:** Association between dominant hand and lateral epicondylitis. Chi-square = 6.448; p = 0.018

		Lateral epicondylitis		Total
		Right	Left	
Dominant	Right	13	5	18
	Left	4	7	11
Total		17	12	29

The mean value of elbow carrying angle in the control group was 9.31 ± 1.93, while it was 13.95 ± 1.14 in the lateral epicondylitis group. The significance of the difference between the mean of the control and lateral epicondylitis groups was examined using the T-test in independent groups, and a significant difference was found between the groups (p < 0.001). The elbow carrying angle level of the LE group was significantly higher than that of the control group (Table 2).

**Table 2:** Assessment of ECA values between groups. ECA: elbow carrying angle, LE: lateral epicondylitis

		N	Mean	Standard deviation	p-value
ECA	Control group	36	9.31	1.93	0,000
	LE group	29	13.95	1.14	

When intra- and interobserver correlations were evaluated, we discovered that angle measurements exhibited high interobserver agreement (ICC, 0.93; 95% confidence interval (CI), 0.91-0.95) and intraobserver agreement (ICC, 0.95; 95% CI, 0.92-0.97) (ICC, 0.94; 95% confidence interval, 0.89-0.96).

## Discussion

The possible etiologic factors for lateral epicondylitis include repetitive microtrauma due to overuse, larger magnitude traumas or manual labor, and anatomical factors, although unclear [7]. In some studies [8,9], ECA abnormalities have been related to diseases such as medial/lateral epicondylitis and ulnar nerve neuropathies; however, some studies have found no relation between ECA and LE [5,6]. The most relevant finding of this study is that increased elbow carrying angles measured radiographically are associated with lateral epicondylitis, although we know the pathogenesis of lateral epicondylitis is multifactorial.

When studies on the etiology of LE were detailed, anatomical factors such as anterior translation of the radial head and lateralization of the extensor carpi radialis longus (ECRL) were identified as contributing to an increase in the pressure on the ECRB between the capitellum and itself [3]. The origin of the ECRL is more proximal to the humerus than the origin of the ECRB, and its course does not cross the forearm rotation axis similar to ECRB. This is considered to be one of the rare instances of ECRL being involved in the pathophysiology of LE [10].

Additionally, Shaaban et al. demonstrated that when elbow flexion degree increases, pronation degree decreases, which may account for the loss of shoulder internal rotation contribution [11]. This results in increased strain on the extensor-pronator muscles to sustain functional pronation. According to Lucado et al., LE is associated with an increase in pronation and a loss of wrist flexion in symptomatic recreational female tennis players. Also, significant loss in shoulder internal rotation is noted for both symptomatic and asymptomatic tennis player groups compared to control [6].

Erickson et al. evaluated elbow carrying angles and upper extremity injuries in professional baseball players and found no significant difference in elbow carrying angles between the injured and non-damaged groups. There was a significant difference in the carrying angles of throwing arms (12.5° ± 4.2°) and non-throwing arms (9.9° ± 2.8°), and five of nine injured players sustained UCL tears [5].

The carrying angle is neutral at full pronation, and according to the radian for the arc length of forearm axial rotation from full supination to pronation, a greater radian will result in a greater distance. The length of the ECRB tendon with the osteotendinous junction site is just under 2 cm with a 0.12 cm difference between male and female subjects [12]. The location and length were found to be consistent also with the study by Cohen et al. [13]. The ECRB origo-musculotendinous junction distance should be considered as the radius of the mentioned rotation because it is the center of the pathology and does not seem to be affected significantly by forearm length or gender. An increase in the arc length will result in higher tension values especially in the limits of ROM according to the Blix curve [14]. In addition, the change of valgus angle into a relatively varus orientation creates varus stress load, which is a significant contact pressure increasing factor [15]. The carrying angle is the main motion-dependent variable that changes the tendon tension, abrasive, and pressurizing forces on the ECRB tendon.

Numerous studies on the relationship between LE and the dominant side have been conducted, with the majority of studies indicating that the dominant side was more affected [16,17]; others indicated the contrary [18]. There was a significant correlation between LE and the dominant side in this study. This result was also consistent with our hypothesis, as it is well established that the carrying angle is greater on the dominant side [19,20].

The results of this study were consistent with the abovementioned mechano-anatomical theory. This theory is derived from the preliminary results of an ongoing study by our study group of arc length differences of forearm axial rotations in children with unilateral cubitus varus deformity without length discrepancy as a complication of supracondylar humerus fractures compared to the unaffected side.

The major limitations of this study were its retrospective design and relatively small study group. Additionally, relying on radiological measurements may affect carrying angle values, as they are altered by elbow flexion/extension.

## Conclusions

Increased elbow carrying angle is associated with lateral epicondylitis and may contribute to its etiology by raising ECRB tendon tension and altering its course, resulting in increased abrasive and pressurizing forces.
